# The UBR5 protein facilitates mesangial cell hypertrophy and glycolysis induced by high glucose by increasing the phosphorylation levels of AKT

**DOI:** 10.1007/s00592-025-02464-9

**Published:** 2025-02-13

**Authors:** Lin Liao, Qiming Xu, Jie Xu, Jie Chen, Wenrui Liu, Wenhao Chen, Yunqing Tang, Lianxiang Duan, Yue Guo, Ziyang Liu, Pengyu Tao, Yu Cao, Jianrao Lu, Jing Hu

**Affiliations:** 1 Department of Nephrology, Seventh People’s Hospital, Shanghai University of Traditional Chinese Medicine, Shanghai, 200137 China; 2 Shanghai University of Traditional Chinese Medicine, Shanghai, 200137 China; 3 Department of Pharmacy, Seventh People’s Hospital, Shanghai University of Traditional Chinese Medicine, Shanghai, 200137 China; 4 Department of Nephrology, Seventh People’s Hospital, Shanghai University of Traditional Chinese Medicine, 358 Datong Road, Pudong New Area, Shanghai, 200137 China

**Keywords:** High glucose, Hypertrophy, Glycolysis, UBR5, AKT phosphorylation

## Abstract

**Aims:**

One of the primary pathological features in the early stages of diabetic nephropathy is mesangial cell (MC) hypertrophy in the glomerulus. Considering the role of E3 ubiquitin ligases in regulating MC hypertrophy, the aim of this study was to identify the functional ubiquitin protein ligase E3 component N-recognin 5 (UBR5) during MC hypertrophy under high glucose conditions.

**Methods:**

Human MCs (HMCs) transduced with UBR5 silencing or overexpression vector were treated with high glucose, AKT inhibitor, or glycolysis inhibitor. Cell proliferation, cell cycle, hypertrophy and glycolysis were evaluated in the HMCs after indicated treatment. m6A methylated RNA immunoprecipitation, luciferase reporter assay, and RNA immunoprecipitation were performed to determine the regulation of UBR5 by Wilms tumor 1-associating protein (WTAP)/insulin-like growth factor 2 mRNA-binding protein 1 (IGF2BP1) induced m6A modification. Western blot was performed to determine the protein expression levels.

**Results:**

UBR5 expression was upregulated in db/db mice and in high glucose-induced HMCs. UBR5 silencing inhibited high glucose-induced HMC cell cycle arrest, cell hypertrophy, and glycolysis. UBR5 facilitated HMC hypertrophy and glycolysis by promoting the phosphorylation levels of AKT. Additionally, the promoting effect of glycolysis on cell hypertrophy were also elucidated. Further investigation into upstream regulators revealed that WTAP promoted m6A modification of UBR5 through the m6A reader IGF2BP1.

**Conclusions:**

Our study unveils a novel mechanism involved in high glucose-induced cell hypertrophy, offering new insights into the understanding and treatment of early pathological mechanisms in diabetic nephropathy.

**Supplementary Information:**

The online version contains supplementary material available at 10.1007/s00592-025-02464-9.

## Introduction

Diabetic nephropathy, which is a major cause of end-stage renal disease, is one of the most critical microvascular complications of diabetes and affects around 40% of diabetic patients [1]. Diabetic nephropathy is characterized by persistent proteinuria, glomerular injury, and renal fibrosis [2]. Our understanding of its complex pathophysiology is very limited. A comprehensive understanding of the complex signaling pathways involved in the pathogenesis of diabetic nephropathy is necessary for effectively understanding its clinical impact. One of the key pathologies of diabetic nephropathy is glomerular lesions, with alterations involving various glomerular cells, including mesangial cells (MCs) [3]. MC hypertrophy is one of the initial pathological anomalies in diabetic nephropathy and leads to increased glomerular filtration rate and microalbuminuria, and gradually progresses to thickening of the basement membrane. Mesangial dysfunction and subsequent glomerulosclerosis are the consequences of MC hypertrophy [4]. Thus, the early hypertrophic changes in MCs ultimately lead to the subsequent development of more severe pathological alterations in diabetic nephropathy.

The pathogenesis of diabetic nephropathy is closely associated with the disorders of glucose and lipid metabolism, among which abnormal glycolysis is the main cause of diabetic renal fibrosis [5]. Clinical data have demonstrated elevated oxygen consumption, renal lactate levels, and glycolysis rates in patients with diabetic nephropathy [6]. Glucose consumption is thought to be promoted by an overexpression of glucose transporter proteins (such as GLUTs). GLUT1 has a high affinity for glucose and is significantly overexpressed in MCs exposed to high glucose conditions [7].

Phosphatidylinositol 3-kinase (PI3K) and its downstream effector serine/threonine protein kinase B (AKT) are the two components of the PI3K/AKT signaling pathway [8, 9], which is activated through phosphorylation, leading to a series of downstream cascade reactions and interactions between target proteins [10]. The aberrant activation of this pathway is not only associated with tumorigenesis but also diabetic nephropathy [11–13]. The phosphorylation of AKT (p-AKT) has been demonstrated to promote cell hypertrophy [14] and GLUT1-mediated glycolysis [15]. Thus, delaying the process of aerobic glycolysis by inhibiting the p-AKT might provide significant therapeutic benefits in the treatment of diabetic nephropathy.

Ubiquitination, which is mediated by ubiquitin ligases effectively influences protein expression [16]. Few previously conducted studies have demonstrated that dysfunction of E3 ubiquitin ligases plays a role in diabetic nephropathy by regulating MC fibrosis, collagen synthesis, and autophagy [17–19]. The ubiquitin protein ligase E3 component N-recognin 5 (UBR5) is a nuclear phosphoprotein that was initially discovered during the screening of progesterone regulatory genes in breast cancer cells [20]. Dysregulated UBR5 is functionally akin to oncogenic proteins and can promote cancer growth, glycolysis, and radiosensitivity via the PI3K/AKT signaling pathway [11, 12, 21]. UBR5 plays a positive role in anabolism/hypertrophy in recovery from skeletal muscle atrophy [22]. UBR5 being an E3 ubiquitin ligase has diverse substrates, and is involved in several physiological and pathological events. However, its role in MC hypertrophy and glycolysis is currently unknown.

N-6-methyladenosine (m6A) methylation is the most common modification that occurs on the RNA in eukaryotes. The interplay between its “writers,” “erasers” and “readers,” governs the abundance and function of m6A in RNA. The variety of proteins constituting m6A writers include methyltransferase-like 3 (METTL3), METTL14, Wilm’s tumor-associated protein (WTAP), RNA-binding motif protein 15, and vir-like m6A methyl-transferase associated (VIRMA) [23]. High glucose condition has been previously reported to regulate m6A writers such as WTAP to affect diabetic nephropathy, thereby promoting m6A abundance [24]. Intriguingly, in head and neck squamous cell carcinoma, UBR5 can be regulated by VIRMA-mediated m6A modification [25]. Hence, we conjectured that WTAP possibly affects the m6A methylation of UBR5 mRNA to regulate its expression in diabetic nephropathy.

In the present study, we aimed to provide novel directions for therapeutic targets in diabetic nephropathy by exploring the potential regulators of MC hypertrophy. Our findings showed the specific molecular mechanisms by which WTAP regulates UBR5 m6A methylation, along with its relationship with the p-AKT, hypertrophy, and glycolysis.

## Materials and methods

### Mouse model of diabetic nephropathy

The Ethics Committee of Shanghai Seventh People’s Hospital (2024-7th-HIRB-097) granted approval for the animal experiment. Six male C57BLKS/J db/db and six male C57BLKS/J db/m mice (8-week-old) were procured from Nanjing Junke Bioengineering Co., Ltd. (Nanjing, China). db/db mice often serve as a common animal model for spontaneous type 2 diabetes because of its leptin receptor deficiency [26]. All mice were maintained in an SPF-grade environment with a temperature of 22 °C ± 2 °C, a relative humidity of 50% ± 5%, and a 12 h light/dark cycle. Serum, urine and kidney tissue samples were collected at the age of 16 weeks. Serum creatinine, blood urea nitrogen, and urine protein levels were measured using the Creatinine Assay Kit, Urea Assay Kit, and Urine Protein Test Kit respectively (all from Nanjing Jiancheng Bioengineering Institute). The kidney tissues were fixed in 4% paraformaldehyde and embedded in paraffin. Section (5 μm) stained with hematoxylin and eosin (H&E) and immunofluorescence staining were observed under microscope for detecting renal histopathological changes and expression of PDGFR-β and UBR5 in kidney tissues. Sections were incubated with anti-PDGFR-β antibody (3169T; Cell Signaling Technology) or anti-UBR5 antibody (66937-1-Ig; Proteintech) followed by the secondary antibody. The percentage of positively stained cells was analyzed. Primary mouse MCs (MMCs) were obtained from freshly harvested glomeruli from db/m and db/db mice and cultured in vitro as previously described [27]. Roswell Park Memorial Institute medium 1640 medium supplemented with 10% fetal bovine serum was used for maintaining the MMCs. 5–7 passages were used for experiments.

## Cell culture

Human MCs (HMCs), procured from ScienCell (San Diego, California, USA) were cultured in DMEM medium supplemented with 10% fetal bovine serum (Invitrogen), penicillin (100 U/ml), and streptomycin (100 µg/ml). For the experiments, cells were cultured in a serum-free medium for 24 h before supplementing the medium with 25 mM glucose for 48 h [28]. For osmotic control, 5 mM glucose and 20 mM mannitol (NG) were used. Otherwise, cells transduced with UBR5 overexpression vector were treated with ATK signaling inhibitor MK-2206 or glycolysis inhibitor 2-DG for 24 h. To determine the optimal concentration of MK-2206 required for treating HMCs under normal glucose condition, different concentrations (0, 2.5, 5, 10, and 20 µM) of MK-2206 were used to treat HMCs for 24 h. For confirming the optimal concentration of 2-DG for treating HMCs under normal glucose conditions, different concentrations of 2-DG (0, 1.25, 2.5, 5, and 10 mM) were tested for treating HMCs for 24 h.

## Gene knockdown and overexpression

Sangon Biotech (Shanghai, China) customized UBR5 shRNA, WTAP shRNA, IGF2BP1 shRNA, and scrambled shRNA sequences, and subcloned them into plasmid vector pLKO.1 (Addgene, Watertown, MA, USA). For lentivirus generation, transfection reagent Lipofectamine 2000 (Invitrogen) was used to transfect shRNA and lentiviral packaging plasmid pMD2. G (paired with plasmid pSPAX2) into 293T cells in accordance with the manufacturer’s instructions. Full-length UBR5 and WTAP were cloned into the pLVX-Puro vector (Clontech Laboratories, Inc., Mountain View, CA, USA) and imported into 293T cells for ectopic expression of UBR5 and WTAP, as mentioned above. The transfected viruses were used to infect the HMCs. Scrambled shRNA (shNC) and empty pLVX-Puro vector (vector) served as controls.

## Cell proliferation analysis

HMCs were inoculated into 96-well plates with 2 × 10^4^ cells/well and treated in accordance with the research design. Then 10 µl cell counting kit-8 (CCK-8) was added to each well and incubated for 2 h away from light. Absorbance was examined at 450 nm with a microplate reader, which was applied to evaluate the proliferation capacity.

## Cell cycle analysis

Infected cells were centrifuged (1000 × g, 5 min), fixed with pre-chilled absolute ethanol, and treated with RNase A to get rid of the RNA interference. Subsequently, 50 µg/mL propidium iodide (PI) was used to stain cells, and flow cytometry (Accuri™ C6, BD Biosciences) was performed to assess cell cycle status.

### Protein synthesis

Cells were incubated with [^35^S]-methionine and then protein synthesis was assessed as previously described [29].

## Cell hypertrophy

After trypsinizing the cells and counting them in a hemocytometer, they were centrifuged at 4000 ×g at 4 °C, and were lysed in RIPA buffer after washing 1X with PBS. The bicinchoninic acid (BCA) Protein Assay Kit (Beyotime) was used to measure the protein content. Hypertrophy was determined in terms of the ratio of total cellular protein to the cell number [29].

## Extracellular acidification rate (ECAR) measurement

The ECAR of cells was monitored using Seahorse XFe24 analyzer (Agilent, Beijing, China) in real-time. After inoculating cells into Seahorse XF cell culture plates, they were gradually treated with glucose, oligomycin, and 2-DG at the time points for ECAR measurement. After replacing the culture medium with the detection medium, the cell culture plate was placed into the Seahorse instrument to detect ECAR.

### Lactate measurement

The levels of the released lactate were measured using a lactate assay kit (A019-2-1, Nanjing Jiancheng Bioengineering Institute). Using PBS as a homogenizing medium, the cultured cells were crushed and centrifuged. The supernatant solution was treated with the enzyme working solution and chromogenic agent and incubated at 37℃ for 10 min. Finally, the absorbance was measured at 530 nm using a microplate reader.

### Real-time quantitative PCR (RT-qPCR)

TRIzol (Invitrogen, Carlsbad, California, USA) was used for extracting the total RNA in HMCs. Oligo (dT) and cDNA synthesis kit (Takara, Japan) were used for the reverse transcription synthesis of cDNA. Maxima SYBR Green qPCR Master Mixes (Thermo Fisher) and a QPCR instrument (ABI 7300, Thermo Fisher) was used to perform RT-qPCR, using the cDNA of the tested gene as a template. Specific forward and reverse primers for UBR5, WTAP, IGF2BP1, or β-actin were used (UBR5 forward 5’-GCTCAGCTGCTTCTCCTTCT-3’ and reverse 5’-GCTTTCGGTTTTCCTGCTGT-3’; WTAP forward 5’-GTAATGGTAGCTCCTCCCGC-3’ and reverse 5’-ACCCCGCACTGAGTTGATTT-3’; IGF2BP1 forward 5’-GCGATGAAGGCCATCGAAAC-3’ and reverse 5’-AGCTTCATGATGGCTTGCCT-3’; β-actin forward 5’-GTCACCAACTGGGACGACAT-3’ and reverse 5’-TAGCAACGTACATGGCTGGG-3’). β-actin was used for normalization and the relative expression levels were calculated using the 2^−ΔΔCt^ method.

### Western blot

The cells were harvested and lysed in RIPA (radioimmunoprecipitation assay) lysis buffer (Beyotime) with proteinase (Sigma, St. Louis, MO, USA) and phosphatase inhibitor cocktail (Pierce, Rockford, IL, USA). BCA (bicinchoninic acid) method was used to measure protein quantification. The protein sample was separated using SDS-PAGE (sodium dodecyl sulfate-polyacrylamide gel electrophoresis), following which it was transferred to polyvinylidene difluoride membranes (Thermo Fisher Scientific). 1% bovine serum albumin was used for blocking the membranes, following which, the primary antibodies were incubated overnight at 4 °C. The primary antibodies utilized in this study include anti-UBR5 (Cell Signaling Technology, 65344, 1:1000), anti- PDGFR-β (Proteintech, 82943-1-RR, 1:5000), anti-GLUT1 (Abcam, ab150299, 1:200), anti-AKT (Cell Signaling Technology, 9272, 1:1000), anti-p-AKT (Cell Signaling Technology, 9271, 1:1000), anti-WTAP (Cell Signaling Technology, 56501, 1:1000), anti-IGF2BP1 (Abcam, ab290736, 1:1000), and anti-β-actin antibody (Proteintech, 81115-1-RR, 1:5000). After washing the membranes thrice with Tris-buffered saline with 0.1% Tween-20, they were incubated with HRP-labeled (horseradish peroxidase-labeled) secondary antibody (ZSGB-BIO, ZB-2301 and ZB-2305, 1:5000) for 1 h at 37˚C. Finally, an enhanced chemiluminescence system (Bio-Rad Laboratories, Inc.) was used to visualize the specific signals. The band intensity was quantified with Image-Pro Plus 6.0 software.

### Enzyme-linked immunosorbent assay (ELISA) for m6A content analysis

The m6A levels were quantified using an ELISA assay kit (Abcam, Shanghai, China) in accordance with the instructions provided.

### m6A methylated RNA immunoprecipitation (MeRIP) for m6A methylation level

MeRIP was performed as mentioned in a previous study [30]. Total RNA isolated from the cells was incubated with protein A/G magnetic beads conjugated with antibodies (anti-m6A or anti-IgG) in RIP (RNA immunoprecipitation) buffer adding protease inhibitors and RNase inhibitors, and was stored at 4 °C overnight. Subsequently, the RNA was eluted from the antibody and RT-qPCR was performed to quantify the level of m6A-modified UBR5.

### Luciferase reporter assay

The UBR5 3′UTR sequence was cloned into the pGl3 vector. After treating the cells with 25 mM glucose, they were transfected with pGl3-UBR5 3′UTR luciferase reporter plasmid and Renilla luciferase pRL-TK vector using Lipofectamine 2000 (Invitrogen). Luciferase activity was standardized in accordance with the manufacturer’s protocol.

### Measurement of mRNA stability

After incubating the cells with 0.2 mM actinomycin D, samples were collected at 0-, 2-, 4-, and 6-hours post-incubation for total RNA extraction. Using oligo (dT) primers, cDNA was synthesized with reverse transcriptase, and mRNA levels were quantified using RT-qPCR.

### RNA immunoprecipitation (RIP) assay

RIP experiments were performed using the Magna RIP kit (Millipore, Beijing, China), to assess the binding of the proteins and RNA. RIP lysis buffer containing RNase inhibitors and protease inhibitors was used for lysing the HMCs. After conjugating the RNA magnetic beads with anti-IGF2BP1 antibody (Abcam, ab184305), they were incubated with the RIP lysate. Anti-IgG antibody (Abcam, ab172730) was used as a negative control.

### Statistical analysis

GraphPad Prism software (version 8.4.2) was used for the statistical analysis. Quantitative data has been presented as mean ± standard deviation. The t-test was used to analyze the difference between two groups of samples, while one-way ANOVA followed by Tukey’s post-hoc test was used if more than two groups were involved.

## Results

### UBR5 expression is upregualted in mouse model of diabetic nephropathy

**Fig. 1:**
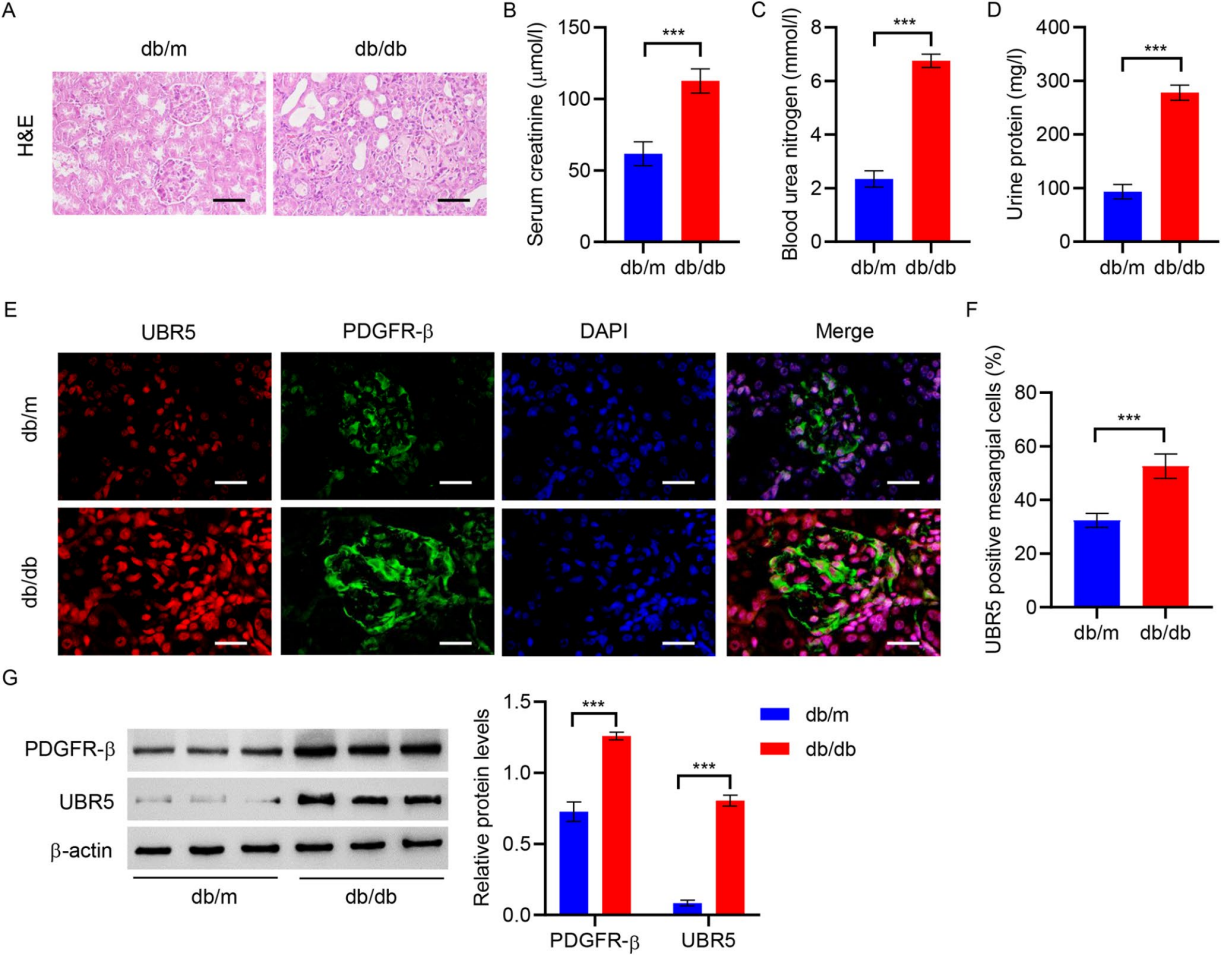
UBR5 expression is upregualted in mouse model of diabetic nephropathy. **A** H&E staining was performed to analyze the pathological changes in renal tissues in db/m and db/db mice (scale bar, 50 μm). Biochemical detection of **B** creatinine, **C** urea nitrogen and **D** urinary protein. **E**,** F** Immunofluorescence staining for PDGFR-β and UBR5 in kidney tissues (scale bar, 20 μm). **G** Western blotting was used to determine PDGFR-β and UBR5 expression in primary MMCs isolated from db/m and db/db mice. ***P < 0.001

A diabetic nephropathy model was established using db/db mice to investigate the role of UBR5 in diabetic nephropathy. H&E staining was performed to observe the renal pathological alterations. The renal tissues of the db/m mice exhibited normal glomeruli with clear tubular structure; however, those of the db/db mice showed dilated lumen of the renal tubules and enlarged glomeruli (Fig. 1A). The db/db mice had significantly elevated levels of serum creatinine, urea nitrogen, and urinary protein compared with the db/m mice (Fig. 1B and D). Immunofluorescence staining was perforemd to analyze the expression of UBR5 in kidney tissues in vivo. As shown in Fig. 1E, the expression of UBR5 in renal glomeruli and tubules was increased in the db/db mice compared with the db/m mice. To further examine the expression of UBR5 in mesangial cells in vivo. Immunofluorescence staining was perforemd to analyze the expression of PDGFR-β, a mesangial cell marker [31], in kidney tissues of db/m and db/db mice. As shown in Fig. 1E and F, the expression of UBR5 in mesangial cells was increased in the db/db mice compared with the db/m mice. Furthermore, the expression of PDGFR-β and UBR5 was also measured in the primary MMCs isolated from db/m and db/db mice. As shown in Fig. 1G, the expression of PDGFR-β and UBR5 was significantly upregulated in the db/db mice compared with the db/m mice.

### UBR5 promotes hypertrophy and glycolysis induced by HG

**Fig. 2:**
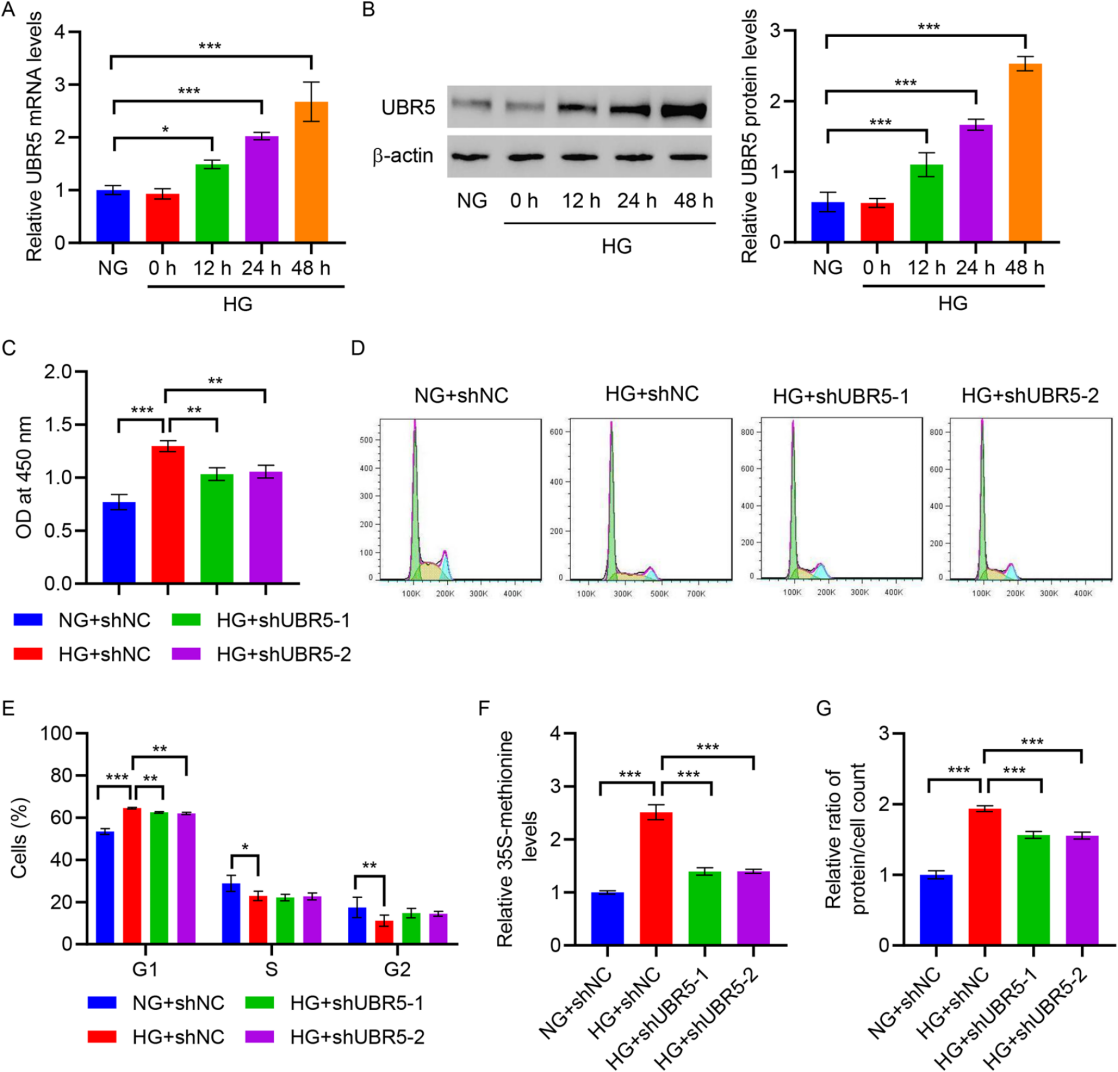
UBR5 knockdown inhibits HG-induced HMC hypertrophy. HMCs were treated with HG (25 mM glucose) for 0, 12, 24, and 48 h, and expression of UBR5 was measured by **A** RT-qPCR and **B** Western blot. **C–G** HMCs were transduced with UBR5-interfering lentivirus and treated with HG (25 mM glucose) for 48 h. **C** Cell proliferation was measured by the CCK-8 assay. **D**,** E** Flow cytometry was performed to examine the cell cycle. **F** 35 S-methionine incorporation assay was to measure protein synthesis. **G** The total protein/cell number ratio was determined to evaluate cell hypertrophy. *P < 0.05, **P < 0.01, ***P < 0.001

To further investigate the role of UBR5 in diabetic nephropathy, the expression of UBR5 was also measured in HMCs treated with HG (25 mM glucose) for 0, 12, 24, and 48 h using RT-qPCR and Western blot. Figure 2A and B illustrate a time-dependent increase in UBR5 expression following HG treatment. For understanding the role of UBR5 in HG-induced changes in HMCs, UBR5 was knocked down using targeted shRNA (Figures S1A and S1B), in addition to the examination of cell proliferation and cell cycle alterations. The results demonstrated a noticeable increase in cell proliferation and the proportion of cells in G1 phase, and decrease in the proportion of cells in S and G2 phases, caused by the HG treatment. However, knockdown of UBR5 reversed the effects of HG treatment on cell proliferation and G1 cell cycle arrest but not affected the proportion of cells in S and G2 phases (Fig. 2C and E). Furthermore, HG treatment induced a noticeable increase in the protein synthesis and cell hypertrophy, which was demonstrated by the increased ratio of protein and cell count, which in turn was significantly inhibited by the knockdown of UBR5 (Fig. 2F and G).

**Fig. 3:**
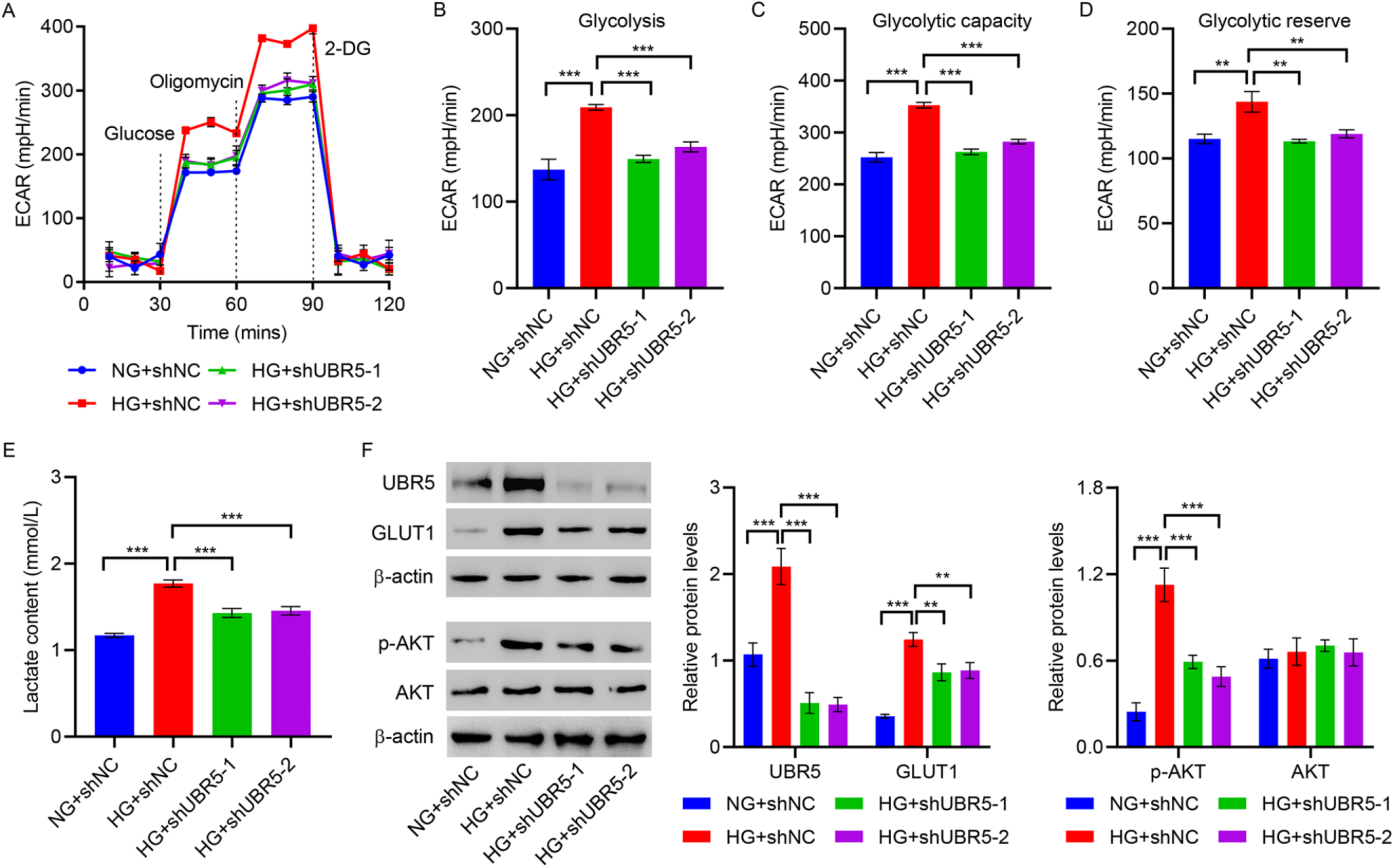
UBR5 knockdown inhibits HG-induced HMC glycolysis. **A–F** HMCs were transduced with UBR5-interfering lentivirus and treated with HG (25 mM glucose) for 48 h. Seahorse energy analyzer was used to assess the **A** ECAR, **B** glycolysis, **C** glycolysis capacity, and **D** glycolytic reserve. **E** Biochemical analysis was performed to identify lactate content in the cell supernatant. **F** Western blot was utilized to evaluate the p-AKT level and expression of UBR5, GLUT1, and AKT. **P < 0.01, ***P < 0.001

To comprehend the potential role of UBR5 in glycolysis, the ECAR and lactate content in the cell supernatant were assessed with the variations in the UBR5 levels. A noticeable increase was observed in ECAR, glycolysis, glycolysis capacity, glycolytic reserve, and lactate content in HG-induced HMCs. However, the HG-induced effects were inhibited by the knockdown of UBR5 (Fig. 3A and E). This indicates that HG induces cellular glycolytic metabolism by promoting UBR5 expression. The p-AKT represents the degree of AKT activation. Glucose transporters are indispensable in the process of glucose metabolism. To further elucidate the underlying mechanism, the level of p-AKT and expression of GLUT1, UBR5, and AKT were analyzed using Western blot. Results demonstrated elevated level of p-AKT and the expression of GLUT1 and UBR5 after HG treatment, which were lowered after UBR5 knockdown (Fig. 3F). However, neither HG treatment nor UBR5 knockdown affected AKT expression.

#### UBR5 facilitates hypertrophy and glycolysis by increasing the p-AKT level

**Fig. 4:**
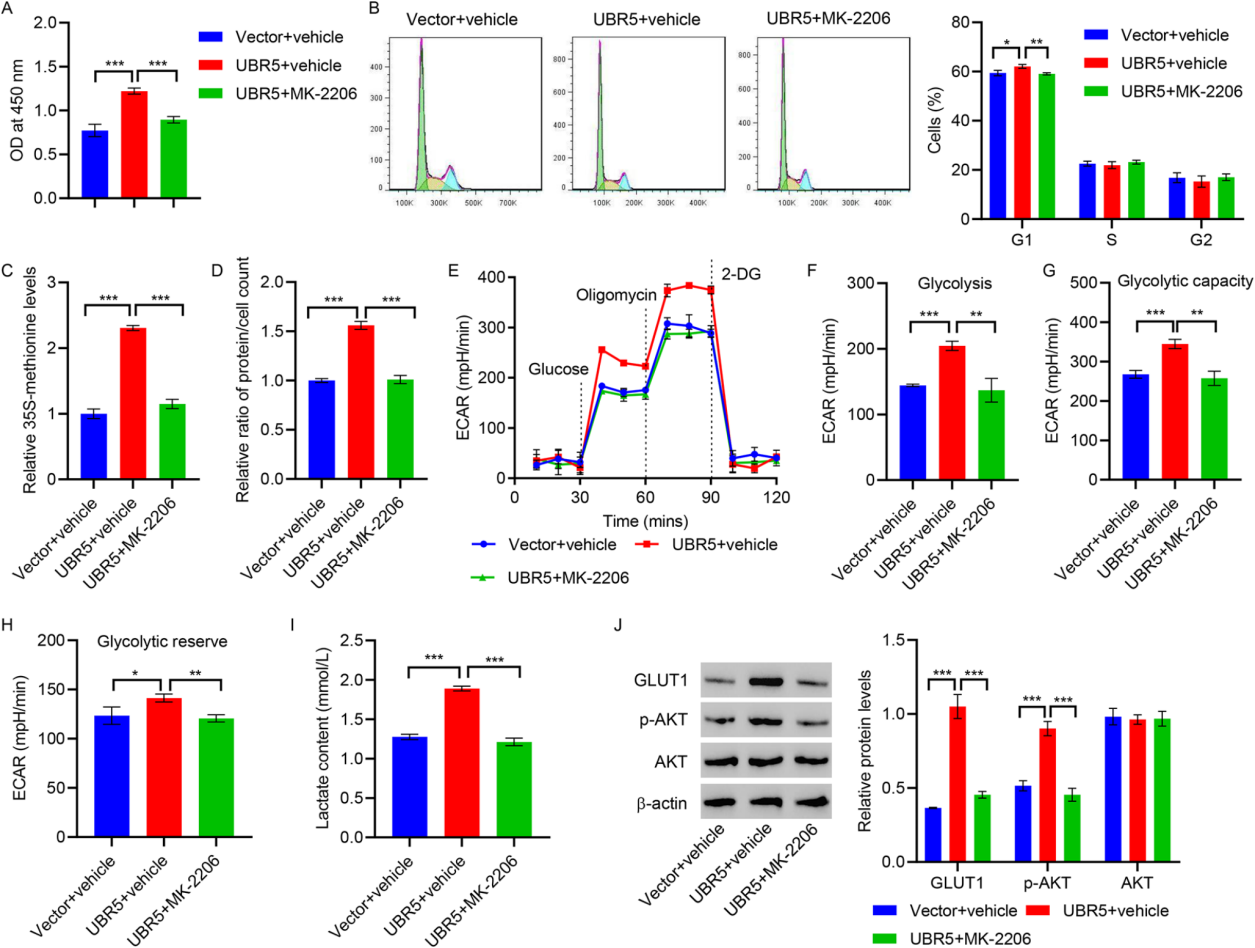
UBR5 overexpression promotes HMCs hypertrophy and glycolysis by promoting p-AKT. HMCs were first transduced with lentivirus overexpressing UBR5 for 24 h, followed by treatment with the AKT inhibitor MK-2206 for an additional 24 h. **A** Cell proliferation was measured by performing the CCK-8 assay. **B** Flow cytometry was performed to examine the cell cycle. **C** 35 S-methionine incorporation assay was conducted to measure protein synthesis. **D** The total protein/cell number ratio was determined to evaluate cell hypertrophy. Seahorse energy analyzer was employed to assess the **E** ECAR, **F** glycolysis, **G** glycolysis capacity, **H** glycolytic reserve. **I** Biochemical analysis was performed to determine the lactate content in the cell supernatant. **J** Western blot was utilized to evaluate the p-AKT level and expression of AKT and GLUT1. *P < 0.05, **P < 0.01, ***P < 0.001

To elucidate the molecular mechanism by which UBR5 promotes HMC hypertrophy and glycolysis, UBR5 was overexpressed in HMCs. The regulatory and activating effects of UBR5 on the p-AKT level were also investigated under normal glucose conditions. RT-qPCR and Western blot confirmed the successful UBR5 overexpression (Figures S1C and S1D). An increase in cell proliferation and the proportion of cells in G1 phase was caused by the exogenous expression of UBR5 (Fig. 4A and B). Examination of 35 S-methionine incorporation and ratio of protein and cell count demonstrated a significant increase in protein synthesis and cell hypertrophy, caused by UBR5 overexpression (Fig. 4C and D). As evident from Fig. 4E and I, overexpression of UBR5 resulted in an increase in ECAR, glycolysis, glycolysis capacity, glycolytic reserve, and lactate content. Western blot results demonstrated that UBR5 increased the p-AKT level and GLUT1 expression but has no effect on AKT expression (Fig. 4J). To determine the optimal concentration of AKT inhibitor MK-2206 required for treating HMCs under normal glucose condition, different concentrations (0, 2.5, 5, 10, and 20 µM) of MK-2206 were used to treat HMCs for 24 h. The CCK-8 results demonstrated a notable reduction in proliferation in MK-2206-treated HMCs compared with the control group. The proliferation capacity of HMCs was lowest at MK-2206 concentration of 10 µM (Figure S2A). Thus, the optimal concentration of MK-2206 for treating HMCs was determined to be 10 µM. As shown in Fig. 4A and J, MK-2206 inhibited the effects of UBR5 on HMC hypertrophy and glycolysis.

### UBR5 promotes HMC hypertrophy through glycolysis

**Fig. 5:**
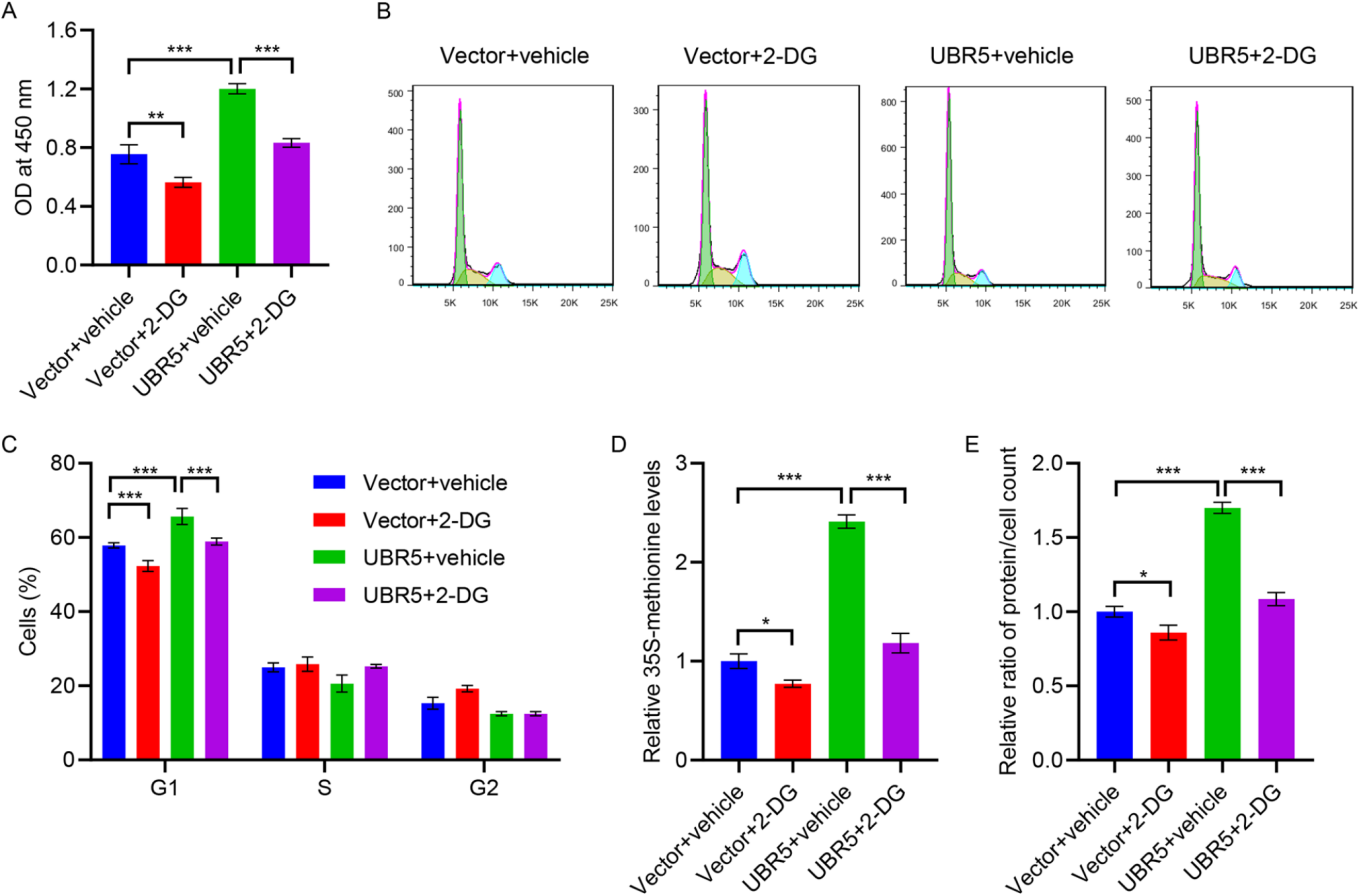
UBR5 overexpression promotes HMCs hypertrophy via glycolysis. HMCs were first transduced with lentivirus overexpressing UBR5 for 24 h, followed by treatment with the glycolytic inhibitor 2-DG for additional 24 h. **A** Cell proliferation was measured by performing the CCK-8 assay. **B**,** C** Cell cycle was assessed by performing flow cytometry. **D** 35 S-methionine incorporation assay was conducted to measure protein synthesis. **E** Hypertrophy was assessed by calculating the ratio of total protein to cell number. *P < 0.05, **P < 0.01, ***P < 0.001

For confirming the optimal concentration of the glycolysis inhibitor 2-DG for treating HMCs under normal glucose conditions, different concentrations of 2-DG (0, 1.25, 2.5, 5, and 10 mM) were tested for treating HMCs for 24 h. The CCK-8 results demonstrated a notable reduction in the proliferation of 2-DG-treated HMCs compared with the control group. The proliferation capacity of HMCs was lowest at 2-DG concentration of 5 mM (Figure S2B). Thus, 5 mM of 2-DG was considered optimal for treating HMCs. As evident from Fig. 5A and E, the effects of UBR5 on HMC proliferation and hypertrophy were inhibited by 2-DG. However, 5 mM of 2-DG did not affect the expression of UBR5 in HMCs (Figure S2C). These findings demonstrate that UBR5 promotes cell hypertrophy by facilitating glycolytic metabolism in HMCs.

#### WTAP promotes UBR5 m6A methylation modification in an IGF2BP1-dependent manner

**Fig. 6:**
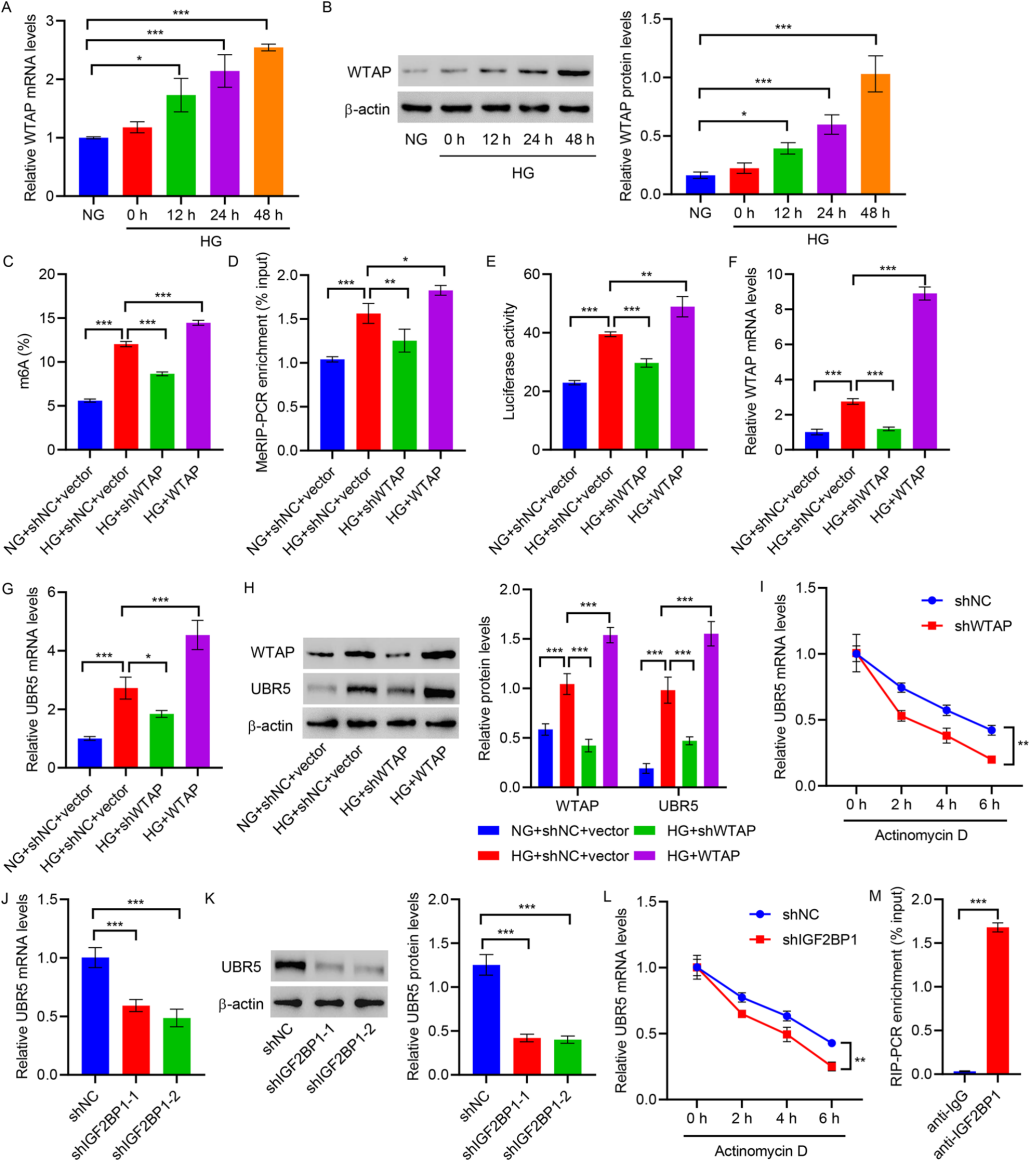
WTAP promotes UBR5 m6A modification in an IGF2BP1 dependent manner. HMCs were treated with HG (25 mM glucose) for 0, 12, 24, and 48 h, and expression of WTAP was measured by **A** RT-qPCR and **B** Western blot. **C–H** HMCs were first transduced with lentiviral vectors for WTAP interference or overexpression and treated with HG (25 mM glucose) for 48 h. **C**,** D** RT-qPCR and **E** Western blotting were conducted to evaluate WTAP and UBR5 expression. **F** Global m6A levels were measured by ELISA. **G** MeRIP-PCR was used to assess UBR5 3’UTR methylation levels. **H** UBR5 3’UTR activity was examined by Luciferase reporter gene assays. **I** HMCs transduced with lentiviral vectors for WTAP interference were treated with 0.2 mM actinomycin D for 0, 2, 4, and 6 h, and RT-qPCR was performed to measure UBR5 transcription levels. HMCs were transduced with lentiviral vectors for IGF2BP1 interference, and **J** RT-qPCR and **K** Western blotting were performed to detect UBR5 expression. **L** HMCs transduced with lentiviral vectors for IGF2BP1 interference were treated with 0.2 mM actinomycin D for 0, 2, 4, and 6 h, and RT-qPCR was performed to measure UBR5 transcription levels. **M** RIP-PCR was executed to detect the binding of IGF2BP1 at the 3’UTR region of UBR5. *P < 0.05, **P < 0.01, ***P < 0.001

Considering the previous findings that indicated a crucial role of methylation in diabetic nephropathy, the regulatory effect of the main component of methyltransferase, WTAP on UBR5 methylation modification was further investigated. RT-qPCR and Western blot revealed a gradual increase in WTAP expression over time following HG stimulation (Fig. 6A and B). RT-qPCR and Western blot were performed to assess the expression of WTAP and UBR5, and indicated that HG treatment promoted the expression of WTAP and UBR5 (Fig. 6C and E, Figures S3A–S3D). WTAP knockdown caused a noticeable reduction in UBR5 expression, while an overexpression of WTAP resulted in the upregulation of UBR5 expression. Moreover, ELISA and MeRIP-PCR demonstrated a significant increase in the global and UBR5 3′UTR methylation levels caused by the HG treatment. The increase in the methylation levels was inhibited by WTAP knockdown and was promoted by WTAP overexpression (Fig. 6F and G). 3’UTR has the ability of modulating protein interactions and gene expression [32]. Luciferase reporter assay revealed a significant enhancement in the activity of the 3’UTR of UBR5, caused by HG treatment. The increase in activity was inhibited by WTAP knockdown and promoted by WTAP overexpression (Fig. 6H). Interestingly, mRNA stability was detected by 0.2 mM actinomycin D treatment, we observed that silencing of WTAP strongly weakened the stability of UBR5 mRNA in HMCs for the subsequent 0.2 mM actinomycin D treatment for six hours (Fig. 6I). All these observations demonstrate that WTAP mediates m6A methylation of UBR5 mRNA to stability this mRNA transcription.

The IGF2BP protein family, act as readers of m6A, and can directly recognize and bind to m6A sites, thereby imparting specific functions to m6A-modified RNAs [33]. In this study, the expression of IGF2BP1 was muted to observe its effect on UBR5 expression. RT-qPCR and Western blot were performed to confirm the successful transduction of shIGF2BP1(Figures S3E and S3F). RT-qPCR and Western blot revealed a significant downregulation in the expression of UBR5 caused by IGF2BP1 knockdown (Fig. 6J and K). Also, silencing of IGF2BP1 strongly weakened the stability of UBR5 mRNA in HMCs for the subsequent 0.2 mM actinomycin D treatment for six hours (Fig. 6L). To test whether IGF2BP1 can directly recognize and bind to the m6A site of UBR5, RIP-PCR analysis was performed, the results of which indicated a significant binding between IGF2BP1 and UBR5 3’UTR (Fig. 6M). This suggests that the m6A methylation modification of UBR5 mRNA by WTAP relies on the recognition and binding of the modification site by IGF2BP1.

## Discussion

Signal transduction and metabolic regulation are intricately intertwined, and their normal interplay is significant for ensuring cellular functional stability and maintaining cellular energy homeostasis [34, 35]. Glucose metabolism is a vital metabolic pathway within cells, and its dysregulation could lead to the onset of diabetes [36]. Elucidating the signaling pathways and regulatory mechanisms of glucose metabolism are necessary for identifying novel therapies for diabetes. Our study is the first one to demonstrate that UBR5 can regulate the level of p-AKT induced by HG, thereby enhancing HMC hypertrophy and glycolysis. Moreover, HG conditions also promote UBR5 m6A modification by increasing the expression of WTAP.

Cell cycle is controlled by various protein kinases, such as cyclins and cyclin-dependent kinases, and progresses continuously from quiescence (G0) to G1, S, G2, and M phases. Cells increase protein synthesis during the G1 phase to prepare for DNA replication, which leads to cell hypertrophy [37]. Most renal cells in mature kidneys are in the G0 phase under normal conditions [37, 38]. However, in diabetic conditions, MCs and podocytes are prompted to enter the cell cycle actively. They later stall in the G1 phase and are accompanied by cell hypertrophy [39]. Earlier studies have demonstrated that HG conditions alter the expression of cell cycle proteins and cyclin-dependent kinase inhibitors, including p27 Kip1 and p21 cip1, causing an arrest at the G1 phase and hypertrophy of renal cells [40]. Previous studies have highlighted that dysfunction of E3 ubiquitin ligases regulates MC fibrosis, collagen synthesis, and autophagy, thereby contributing to diabetic nephropathy [17–19]. The E3 ubiquitin ligase, UBR5, is positively associated with anabolism/hypertrophy in recovery from skeletal muscle atrophy [22]. Our study demonstrated that UBR5 expression in mesangial cells was increased in the db/db mice and UBR5 knockdown inhibited HG-induced HMC hypertrophy, indicating that UBR5-mediated HMC hypertrophy might play an important role in diabetic nephropathy. Additionally, UBR5 expression in renal tubules was also increased in the db/db mice. Dysfunctional renal tubular epithelial cells, induced by HG, are commonly observed in the kidney tissues of diabetic nephropathy patients [41], and E3 ubiquitin ligases have also been reported to play a crucial role in HG-induced renal tubular injury [42, 43]. These data suggest that UBR5 may also facilitate diabetic nephropathy by inducing renal tubular injury.

Glycolysis, is a fundamental and crucial pathway of glucose metabolism, and provides the necessary energy and intermediate products for the life activities of tissues and cells. Glycolysis generates large quantities of lactate, because of which, ECAR and lactate levels in the cell culture supernatant are considered as crucial indicators for assessing the rate of glycolysis [44]. Activation of specific signaling pathways significantly enhances the activity and expression of the corresponding enzymes, which in turn increases the metabolic flux through glycolysis and related biosynthetic pathways [45]. The molecular mechanisms of glycolysis have been extensively studied in cancer and are considered as potential targets for cancer management [21, 46]. The role played by glycolysis in diabetes and its associated complications have rarely been discussed. Previously conducted studies have highlighted the role of the PI3K/AKT signaling pathway various cellular and life activities, and the PI3K/AKT signaling pathway has been shown to regulate cell proliferation, cell cycle, and apoptosis in diabetic nephropathy [47, 48]. The PI3K/AKT signaling pathway is also involved in regulating MC hypertrophy and matrix expansion induced by HG [14, 49]. The complex structure and function of signaling pathways can be witnessed in their intricate upstream-downstream relationships and their ability to be activated and regulated by various factors while regulating downstream target molecules. Previously conducted studies have demonstrated that the p-AKT promotes cell hypertrophy by increasing mTOR/p70S6K signaling activity [14] and GLUT1-mediated glycolysis [15]. In accordance with the previous studies, induction of p-AKT and promotion of GLUT1 expression resulted from UBR5 overexpression, while the inhibition of p-AKT relieved the UBR5 overexpression induced HMC hypertrophy and glycolysis. One previous study has indicated that UBR5 increased the level of p-AKT by inducing ubiquitination and degradation of SIRT7 [50]. As an E3 ubiquitin ligase, UBR5 is associated with diverse substrates, and has been confirmed to be involved in physiological and pathological events. UBR5 knockdown induced expression of UBR5 in HG condition was even lower than the expression in NG condition, while the GLUT1 expression and levels of p-AKT and lactate release in HG condition were higher than that in NG condition, thereby indicating the existence of other regulators between UBR5 and p-AKT in HMCs. Furthermore, the strong association between the activation of glycolysis and cell growth, including cardiac hypertrophy [51], aligns with our study. In this study, it was observed that the glycolysis inhibitor 2-DG can inhibit UBR5 overexpression-induced HMC hypertrophy, a fact that had not been examined in previous studies.

WTAP is the major component of the methyltransferase complex, and is responsible for forming most of the m6A deposits on mRNA [52]. In this study, a positive association was observed between the expression of WTAP and HG condition, which was consistent with the m6A methylation effect of WTAP. mRNA might possibly be regulated by the RNA methyltransferase WTAP through the m6A modification. WTAP regulates the m6A modification of DKK3, thereby promoting cell proliferation and migration in diabetic nephropathy [53]. WTAP-mediated m6A modification of glycolytic enzyme ENO1 promotes the progression of diabetic nephropathy [54]. m6A modification can directly impact mRNA splicing, translocation, localization, translation, and stability. Recent studies have demonstrated that IGF2BPs, a family of readers, stabilize the translation of target mRNA [33]. IGF2BP1 expression has been reported to be elevated in the mouse model of diabetic nephropathy [55]. Therefore, exploring the molecular mechanisms of IGF2BP1 is crucial for enhancing and advancing our understanding of RNA modification in diabetic nephropathy. In this study, WTAP was observed to be activated under HG stimulation, to increase m6A methylation levels of UBR5, while IGF2BP1 was observed to recognize the corresponding m6A to influence the UBR5 gene expression levels.

Our research is still in its early stages and has some limitations. First, this molecular pathway was verified only at the cellular level without conducting in vivo experiments. Further in vivo exploration and validation are warranted in the future. Second, this study exclusively concentrates on the pathological feature of hypertrophy, while diabetic nephropathy exhibits pathological diversity, affecting all structural components of the kidney. The influence of hypertrophy on the follow-up progress of diabetic nephropathy also warrants further exploration. Third, UBR5 induced HMC hypertrophy through glycolysis; however, its involvement in alternate mechanisms to influence HMC hypertrophy remain unclear. Fourth, UBR5 expression in renal tubular epithelial cells was also increased in the db/db mice. Therefore, its role in HG-renal tubular epithelial cell injury and nephropathy in diabetic db/db mice is not clear. Finally, our focus is limited to the WTAP-mediated m6A modification of UBR5. This study did not investigate the effect of WTAP on HMC hypertrophy and glycolysis under HG conditions.

## Conclusion

In conclusion, our study has elucidated the significant roles played by several proteins in HG-induced HMC hypertrophy, proposing a comprehensive molecular pathway. The HG condition was observed to activate WTAP, thereby enhancing the catalysis of UBR5 m6A methylation modification. IGF2BP1 recognizes and binds to this modified site, subsequently enhancing UBR5 gene expression levels. It thus promotes p-AKT to accelerate cell glycolysis and hypertrophy. The results of this study also emphasize the unexpected role of URB5 in HG-induced HMC hypertrophy and elucidate its potential molecular mechanisms. This regulation pattern highlights new directions for targeted treatment of HMC hypertrophy and provides more effective therapeutic opportunities.

## Electronic supplementary material

Below is the link to the electronic supplementary material.

Supplementary file1 (DOCX 1090 KB)

## Data Availability

On request, information will be made available.
