# Supplementary material for: The UBR5 protein facilitates mesangial cell hypertrophy and glycolysis induced by high glucose by increasing the phosphorylation levels of AKT
Source: Acta Diabetol. 2025 Feb 13;62(9):1403–16. doi: 10.1007/s00592-025-02464-9 (PMC12433351; doi:10.1007/s00592-025-02464-9)
Supplement: Supplementary file 1 — Supplementary file1 (DOCX 1090 KB) [file 592_2025_2464_MOESM1_ESM.docx]

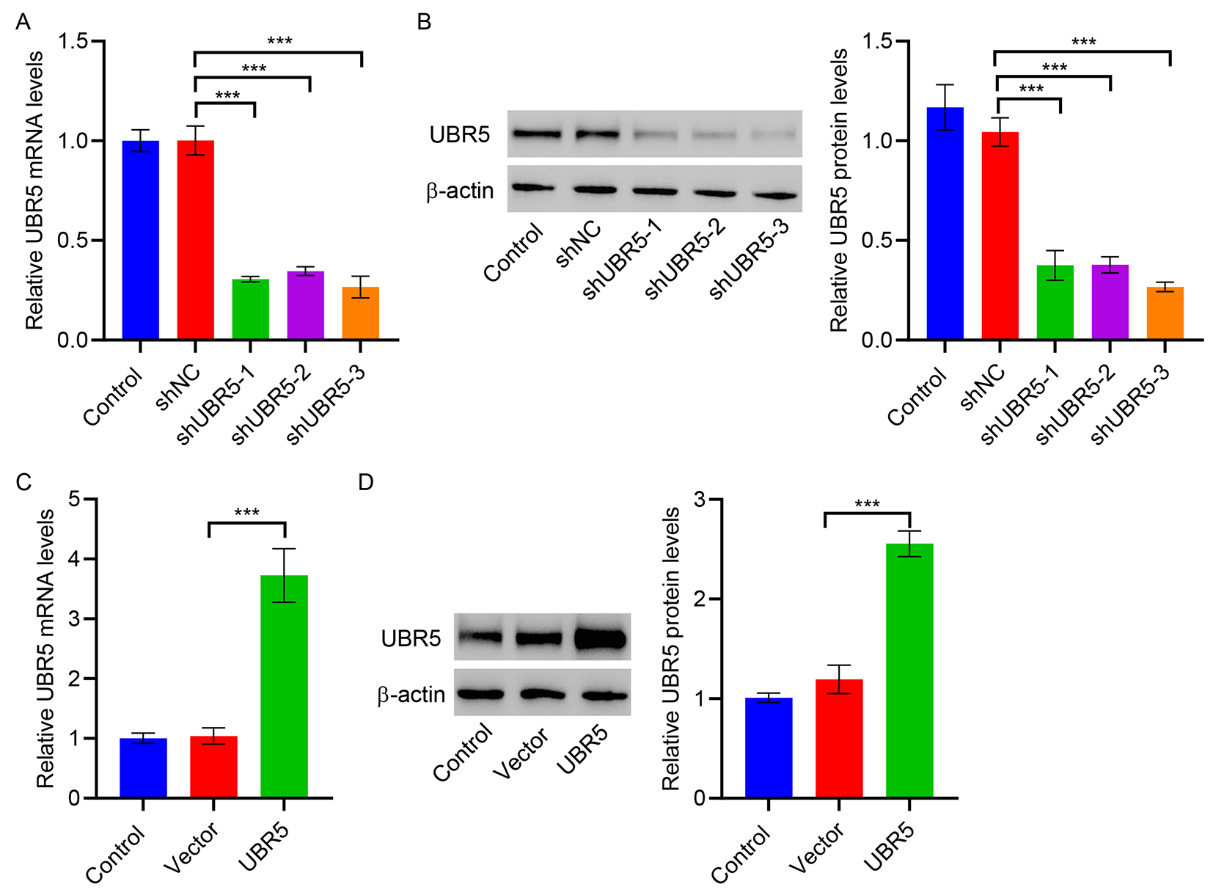


**Figure S1. UBR5 expression in** **HMCs.** Construction of HMCs transduced with (**A, B**) UBR5 gene interference and (**C, D**) overexpression lentivirus; and RT-qPCR and Western blotting were used to detect UBR5 expression. ***P < 0.001.


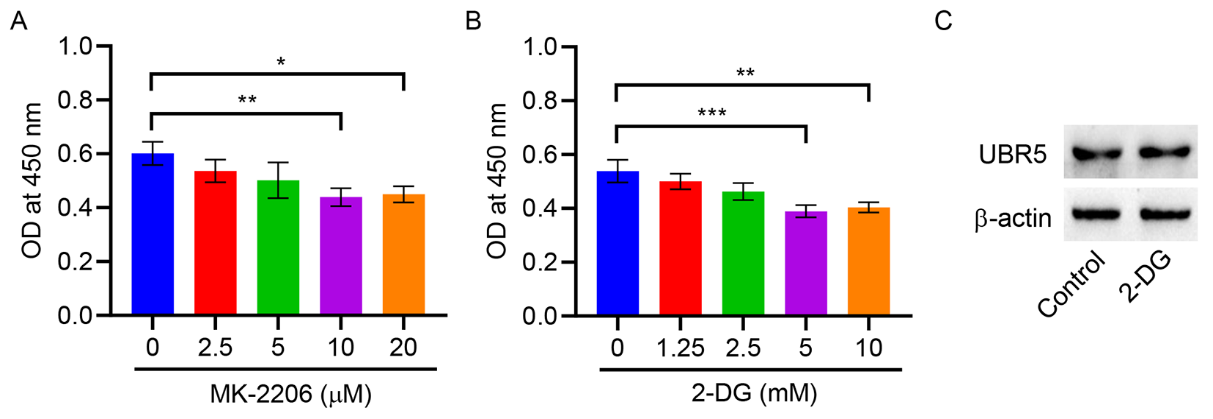


**Figure S2. Screening of appropriate concentrations of MK-2206 and 2-DG in HMCs.** (**A, B**) CCK-8 was performed to confirm the change of cell proliferation in HMCs after treating with 2.5, 5, 10, and 20 μM MK-2206 or 1.25, 2.5, 5, and 10 mM 2-DG for 24 h. (**C**) Western blotting was used to detect UBR5 expression in HMCs after treating with 5 mM 2-DG for 24 h. *P < 0.05, **P < 0.01, ***P < 0.001.


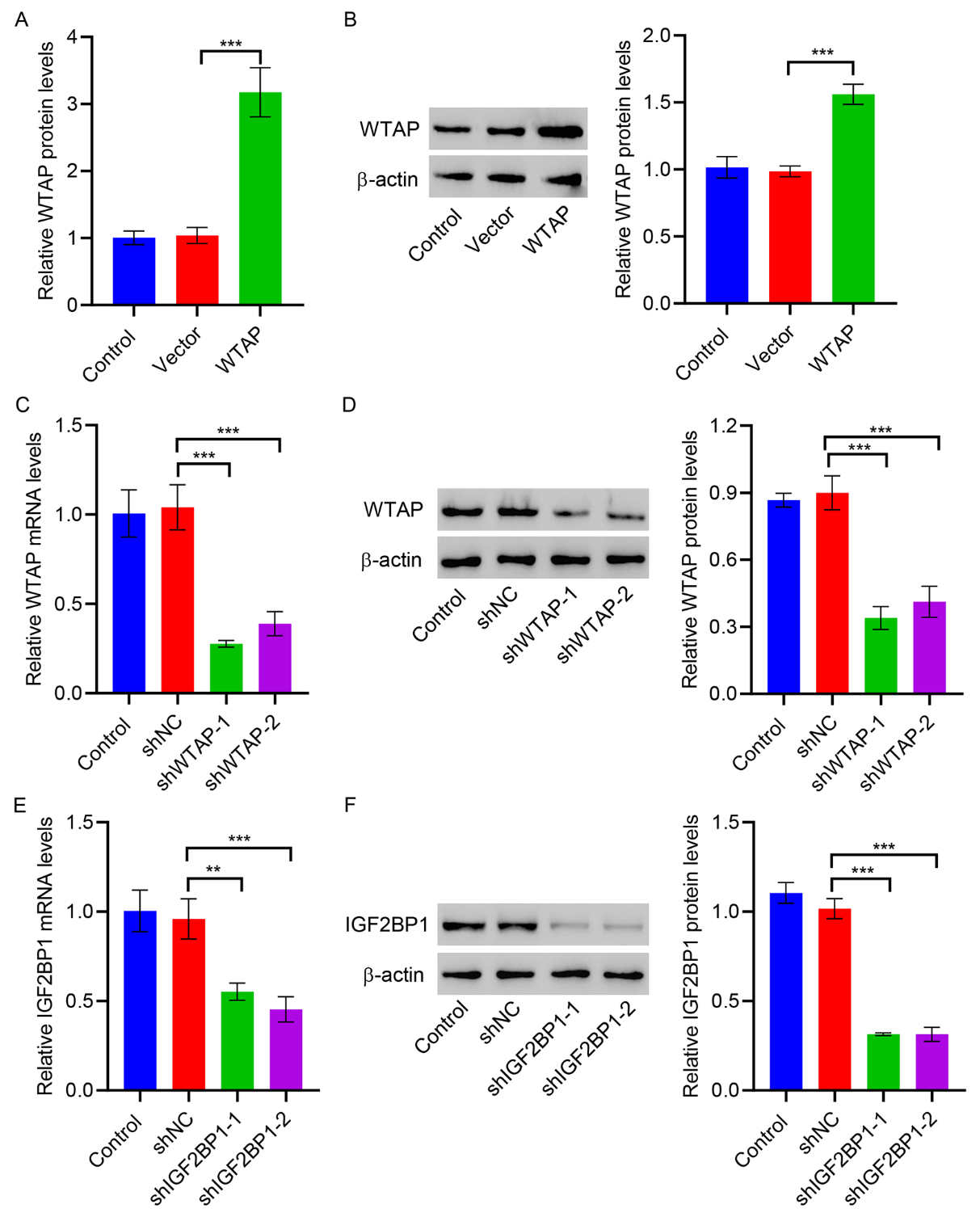


**Figure S3. WTAP and IGF2BP1 expression in** **HMCs.** Construction of HMCs with (**A, B**) WTAP gene overexpression and (**C, D**) interference lentivirus transduction, and RT-qPCR and Western blotting were used to detect WTAP expression. (**E, F**) Construction of HMCs with IGF2BP1 gene interference transduction; RT-qPCR and Western blotting were used to detect IGF2BP1 expression. **P < 0.01, ***P < 0.001.
